# INTERGENERATIONAL SERVICE: EXTENDING AGE INCLUSIVITY IN AND BEYOND THE CLASSROOM

**DOI:** 10.1093/geroni/igae098.1638

**Published:** 2024-12-31

**Authors:** Joann Montepare, Carrie Andreoletti

**Affiliations:** Chair; Discussant

## Abstract

Higher education is becoming more age-inclusive on several fronts and age-friendly teaching and learning strategies are being used in new and novel ways to support a variety of educational outcomes. In this AIHE-ILRCE Interest Group collaborative symposium, presenters will discuss the diverse ways in which intergenerational service activities extend learning beyond the classroom and can enhance age inclusivity in higher education. To begin, Montepare and Baldizar (Lasell University) will provide an overview of contemporary age-inclusive campus initiatives in higher education (Age-Friendly University/AFU, Age Inclusivity Domains of Higher Education/AIDHE) and discuss The Empty Bowls project in which students and older adult community members work together to craft bowls to raise money to support the local Food Pantry. Brothers (Colorado State University) will describe the student-supported Market Days for Older Adults project, a farmer’s market-based program that aspires to address food insecurity and social isolation in older adults. Wei and colleagues (Stockton University) will discuss an intergenerational service project in which students organize technology training sessions aimed at educating older adult community members about various technology-related topics such as using smartphones for chronic disease management, personalized medicine, and more. Kwong (Connecticut College) will describe an interdisciplinary class, Intergenerational Play and Learning, which connects young children, college students, and older adult volunteers to promote learning through storytelling and engaging in play and group activities to strengthen social ties across generations and explore local history. AGHE Fellow and intergenerational scholar, Carrie Andreoletti (Central Connecticut State University), will serve as discussant. Intergenerational Learning, Research, and Community Engagement Interest Group Sponsored Symposium

